# The effect of chelating agents including potassium tartrate and citrate on the maximum reduction of lead and cadmium during soaking and cooking from some different varieties of rice available in Iran

**DOI:** 10.1002/fsn3.2473

**Published:** 2021-07-15

**Authors:** Zahra karimi, Mohammad Goli

**Affiliations:** ^1^ Department of Food Science and Technology Isfahan (Khorasgan) Branch Islamic Azad University Isfahan Iran; ^2^ Laser and Biophotonics in Biotechnologies Research Center Isfahan (Khorasgan) Branch Islamic Azad University Isfahan Iran

**Keywords:** 3‐hr soaking step, 15‐min boiling step, heavy metal removal, Inductively coupled plasma mass spectrometry, sensory evaluation, sequestering agents

## Abstract

This study aimed to determine the percentage of reduction of lead and cadmium by chelating agents (potassium tartrate and potassium citrate) in the steps of soaking, cooking, and simultaneous soaking and cooking in some varieties of rice for the first time. Each chemical experiment was performed in ten replications. Inductively coupled plasma mass spectrometry (Agilent‐7700X ICP‐MS) was used to assess the complete Cd and Pb content in rice samples acid‐digested (500 mg dry‐sample, 9 ml HNO_3_: 3 ml HCl). The cooking‐only treatment was more successful in terms of lead reduction than the soaking‐only treatment in chelating agent‐containing solutions (either potassium tartrate or potassium citrate), though it had the same effect on cadmium reduction. Simultaneous soaking and cooking in chelating agents such as potassium tartrate and potassium citrate significantly reduced lead (reduction rate compared to control 99.43% with potassium tartrate and 98.96% with potassium citrate) and cadmium (reduction rate compared to control 95.13% with potassium tartrate and 92.77% with potassium citrate). Potassium tartrate outperforms potassium citrate in terms of lead reduction, but potassium tartrate is equivalent to potassium citrate in terms of cadmium reduction. Up to 200 ppm applicable chelating agents, sensory analysis showed no statistically significant difference between the treatments. In general, rice cookers are advised to use levels up to 200 ppm of citrate or potassium tartrate in combination in the 3‐hr rinsing period and then in the 15‐min cooking period to reduce the percentage of dangerous heavy metals, especially lead 99%–99.4% and cadmium 92.8%–95.1%.

## Graphical abstract

Cooking than soaking treatments by chelating agents (CAs) caused more Pb reduction. Cooking than soaking treatments including CAs caused to the same Cd reduction. Soaking–cooking treatments including CAs significantly reduced Pb, that is, 99%–99.4%. Soaking–cooking treatments including CAs significantly reduced Cd, that is, 92.8%–95.1%. Potassium tartrate outperforms potassium citrate in terms of lead reduction.

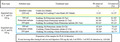

## 1 INTRODUCTION

The increasing gap between population growth and food reserve, especially in developing and underdeveloped regions of the world, has created further interest in finding reputable and inexpensive sources of plant resources with potential nutraceutical and health‐promoting properties (Bhat et al., 2013). Rice, after wheat, is one of the most commonly consumed grains in the world, especially in Asian countries, where it provides approximately 70% of daily calorie requirements. According to estimates, the annual per capita consumption of rice in Iran is approximately 40 kg, whereas the per capita consumption of rice in Asia is 85 kg and in the world is equal to 65 kg (Ziarati & Moslehi‐shad, 2018).

Heavy metals are toxins found in the environment, and human exposure to them by water and food can result in acute, chronic, and life‐threatening poisoning (Shuklasr, 2005). Heavy metal contamination of soil and water is one of the most significant environmental stresses for plants, and these metals can threaten human life across the food chain. Heavy metal pollution of rice (irrigation of contaminated water, insufficient storage or handling of wastewater, use of chemical fertilizers to develop soil properties) is one of the issues that humans are currently dealing with (Chaney et al., 2004). Heavy metal poisoning can lead to complications such as neurological disorders, various cancers, nutrient deficiencies, hormonal imbalances, abortion, respiratory and cardiovascular disorders, liver damage, kidney, brain, allergies, loss of appetite, prematurity, decreased memory, hair loss, osteoporosis, insomnia, weakened immune system, anemia, gene damage, and even death. Severe dietary accumulation of heavy metals such as Pb, Cd, Ni, and Cr can cause serious health problems. Lead and cadmium are thought to be particularly toxic. Kidney injury, as well as bone effects and fractures, are possible side effects of cadmium toxicity. Long‐term exposure to Pb has also been linked to health problems such as memory loss, slower response times, and a decreased capacity to comprehend (Naseri et al., 2014).

The effects of conventional food processing methods on heavy metal levels have been studied in a number of studies. Cooking has been shown to change the concentration or amount of these contaminants in food tissues such as seafood (Appleton et al., 2006), vegetables (Sharma et al., 2007), and rice (Naseri et al., 2014). The cooking method and components of the rice cooking process influence heavy metal contamination. Heavy metals may be reduced to a large degree using such cooking techniques (Morekian et al., 2013). Naseri et al. (2014) investigated at how different cooking processes (Pilaw, or rinsing and Kateh, or unrinsing) affected the concentration of heavy metals (cadmium, lead, chromium, nickel, and cobalt) in imported rice. The results revealed that the rate of metal reduction after cooking differed with each metal, with Co>Ni> Cd>Pb> Cr being the most common. The effect of cooking on the bioavailability of cadmium and arsenic in rice was investigated by Zhuang et al. (2016). Cadmium and arsenic bioavailability in rice was reduced due to the cooking procedure.

In food systems, free metal ions may form insoluble or colored compounds or catalyze the deterioration of food materials, resulting in accumulation, discoloration, rancidity, or nutritional quality loss. Chelating agents, for example, EDTA, potassium tartrates, and potassium citrate, eliminate these negative effects by forming stable, normally water‐soluble complexes of free metal ions. This is known as chelation, and the complexes that form are known as chelates (Nauta, 1991). K_2_C_4_H_4_O_6_ is the formula for potassium tartrate, dipotassium tartrate, and argol. It is tartaric acid's potassium salt. The reaction of tartaric acid with potassium sodium tartrate (rochelle salt) and potassium sulfate results in potassium tartrate, which is then filtered, purified, precipitated, and dried. The potassium salt of citric acid with the molecular formula K_3_C_6_H_5_O_7_ is potassium citrate (also known as tripotassium citrate). It is a crystalline powder that is white and hygroscopic. It has a saline flavor and is odorless. It has a potassium content of 38.28% by mass. It is extremely hygroscopic and deliquescent in its monohydrate shape. Potassium citrate is made by applying potassium bicarbonate or potassium carbonate to a citric acid solution before effervescence stops, then filtering and evaporating to granulation. Yang et al. (2016) investigated methods for removing lead and cadmium from vegetable oil and their meal. Potassium tartrate and potassium citrate effectively reduced the amount of heavy metals in oilseed meal (Yang et al., 2016) and from some different varieties of rice available in Iran (Hashemi garmdareh and Goli, 2020; Hashemi garmdareh and Goli, 2019).

Due to the contamination of imported and exported rice with heavy metals, basic methods such as soaking in plain water have been suggested to eliminate them. Therefore, the aim of this study was to determine the percentage of reduction of lead and cadmium by chelating agents (potassium tartrate and potassium citrate) in the steps of soaking, cooking, and simultaneous soaking and cooking in some types of imported and exported rice in Iran.

## 2 MATERIALS AND METHODS

### 2.1 Materials

Three types of imported rice included codes I, T, U imported from India, Thailand, and USA, respectively, and three types of domestically produced rice included codes A, P, S were purchased from local market (Isfahan, Iran). For each type of rice, 10 samples of 10 kg were prepared. Each chemical experiment was performed in ten replications. Citrate or potassium tartrate (Merck, Germany) concentrations of 50 to 5,000 ppm (standard permeable limit of codex alimentarius, CODEX STAN: 192–1995) were used in the initial experiments (Codex Alimentarius Commission (CAC), 2005). In this study, the best concentration preferred was 200 ppm because concentrations above 200 ppm of these chelating agents not only induced acidification of cooked rice but also did not show a significant difference in reducing rice lead and cadmium.

### 2.2 Preparation of treatments

The volume/weight ratio of soaking or/and cooking solution to rice was 2:1 in all treatments. To make the soaking and cooking solutions, distilled water was used. 100 g of each rice type was soaked for 3 hr in 200 ml of 200 ppm aqueous potassium citrate or tartrate solution before being cooked for 15 min in 200 ml distilled water and 30‐min brewed (S‐Cit, S‐Tar) or before being cooked for 15 min in 200 ml of 200 ppm aqueous potassium citrate or tartrate solution and 30‐min brewed (SC‐Cit, SC‐Tar). All of eight treatments were prepared according to the Table 1 (Hashemi garmdareh and Goli, 2020; Hashemi garmdareh and Goli, 2019).

**TABLE 1:** Treatments used in this study

No	code	Process type
1	blank	Crude sample
2	SC‐blank	Soaking (3 hr) and cooking (15 min) without the presence of chelating agents
3	S‐Tar	Soaking (3 hr) in the presence of potassium tartrate (200 ppm)
4	C‐Tar	Cooking (15 min) in the presence of potassium tartrate (200 ppm)
5	SC‐Tar	Soaking (3 hr) and cooking (15 min) in the presence of potassium tartrate (200 ppm)
6	S‐Cit	Soaking (3 hr) in the presence of potassium citrate (200 ppm)
7	C‐Cit	Cooking (15 min) in the presence of potassium citrate (200 ppm)
8	SC‐Cit	Soaking (3 hr) and cooking (15 min) in the presence of potassium citrate (200 ppm)

Note

The volume/weight ratio of soaking or/and cooking solution to rice was 2:1 in all treatments. To make the soaking and cooking solutions, distilled water was used.

### 2.3 Lead and Cadmium assessment

Acid digestion was used to determine the total Cd and Pb content in rice samples. Total Cd and Pb concentrations [500 mg dry ash, 9 ml HNO_3_ (Merck, Germany): 3 ml HCl (Merck, Germany)] were determined after 20 min in an Ethos Up SK‐12 microwave oven (Milestone INC). After that, the samples were centrifuged and filtered (cellulose acetate membrane filter 0.45 mm). The extract was held refrigerated (4°C) until it was analyzed using an inductively coupled plasma mass spectrometer (Agilent 7700X ICP‐MS). To validate the method of extracting total metals, standards with six different concentrations were prepared from a stock solution of lead (Merck, Germany) and cadmium (Merck, Germany) with a concentration of 1,000 mgL^‐1^, and the corresponding standard curve was drawn. The rate of recovery of Cd and Pb was greater than 95% (Chang et al., 2016; Ochoa et al., 2020).

### 2.4 Sensory evaluation

The cooked‐rinsing rice samples were evaluated using a five‐point hedonic scale. A panel of 20 trained naive consumers (for product scoring and questionnaire completion) and 20 food science and technology students assessed the samples for taste, aftertaste, texture, color, and overall acceptance. The overall acceptance is determined by the average sensory scores obtained from taste, aftertaste, texture, and color. For each sensory property, the value score ranged from 5 (like extremely) to 1 (dislike extremely) (Moslehi et al., 2021; Nazari & Goli, 2017; Zonoubi & Goli, 2021).

### 2.5 Statistical analysis

According to the experimental design, a factorial test was carried out. The SPSS software was used to interpret the results (version 22). Duncan's test was used to quantify important variations at the 5% level of confidence in mean values. Microsoft Excel 2010 was used to build the diagrams (Karshenas et al., 2018; Mei et al., 2020; Parsaei et al., 2018).

## 3 RESULTS AND DISCUSSION

### 3.1 Lead content of imported and exported rice

Table 2 shows the lead content of three types of imported rice and three types of exported rice. There was a statistically significant difference in lead levels between imported rice and exported rice (*p* < .05). The highest and lowest levels of lead were around 1896 and 113 ppb, were confirmed to be around in imported T and I rice, respectively. The highest and lowest levels of lead in exported rice were confirmed to be around 2,390 and 133 ppb, in samples A and P, respectively. Both exported and imported samples had a lead reduction of 70.33–74.17 percent, which was not statistically significant. WHO regulations set the lead limit for rice at 150 ppb (Food and Agricultural Organisation (FAO), 2004). According to some studies, the proximity of rice fields to industrial centers, as well as pollution of water and soil with their wastewater, is a cause of heavy metal accumulation, especially lead, cadmium, and arsenic (Cao and Hu, 2000). Other causes of contaminants of agricultural soils include agricultural chemicals and other human activities (Zhao et al., 2010). In terms of lead, several experiments have shown that soil and plant roots, including rice, can accumulate and stabilize lead. As a result, trace amounts of lead are transferred to rice grains through water and soil. Lead pollution is more common in crops near roads and industrial plants. While there have been evidence of exposure to lead in rice from a variety of sources, there has been no research on the impact of chelating agents in the soaking or/and cooking process on the reduction of lead in rice.

**TABLE 2:** Comparison of lead and cadmium content (ppb) in dried crude rice and them removal‐percent in dried cooked‐rinsing

Rice type		Crude rice (Blank)		Dried cooked‐rinsing rice (soaked or/and cooked with or without chelating agents)	
		Lead ($\frac{\mu g}{\mathrm{Kg}\;\mathrm{dried}\;\mathrm{rice}}$)	Cadmium ($\frac{\mu g}{\mathrm{Kg}\;\mathrm{dried}\;\mathrm{rice}}$)	Lead removal (%)	Cadmium removal (%)
Imported	I	112.92 ± 30.68^b^	30.74 ± 5.67^b^	70.33 ± 8.06^a^	61.60 ± 7.06^a^
	T	1895.60 ± 592.39^a^	33.52 ± 6.06^b^	74.17 ± 8.06^a^	60.99 ± 7.04^a^
	U	538.80 ± 158.64^b^	35.19 ± 5.82^b^	72.73 ± 8.03^a^	59.19 ± 6.74^a^
Exported	A	2,390.40 ± 736.41^a^	177.65 ± 51.23^a^	73.59 ± 8.13^a^	72.06 ± 8.06^a^
	P	133.04 ± 35.94^b^	38.71 ± 7.39^b^	71.86 ± 7.58^a^	63.01 ± 7.05^a^
	S	240.60 ± 64.42^b^	135.06 ± 37.72^a^	70.59 ± 7.87^a^	71.10 ± 8.06^a^

Note

Means ± SE (*n* = 10) with different letters in each column indicate significant difference (*p* < .05). WHO regulations set the lead and cadmium limit for rice at 150 and 60 ppb, respectively.

### 3.2 Cadmium content of imported and exported rice

Table 2 shows the lead content of three types of imported rice and three types of exported rice. There was not a statistically significant difference in cadmium levels between imported rice (*p* > .05). The highest and lowest levels of cadmium in exported rice were confirmed to be around 177.7 and 38.7 ppb, in samples A and P, respectively. Both exported and imported samples had a cadmium reduction of 59.19–72.06 percent, which was not statistically significant. The cadmium limit for rice is set at 60 ppb by WHO regulations (Food and Agricultural Organisation (FAO), 2004). Arsenic accumulation in rice and an arsenic crisis have been identified in studies on crops grown in Bangladesh, India, China, Vietnam, and Indonesia, as well as irrigation of rice with arsenic‐contaminated water and cultivation of rice on contaminated fields. In this respect, one of the causes of cadmium toxicity in rice is contaminated agricultural fertilizer (Morekian et al., 2013). According to Ke et al. (2015), the amount of cadmium in 484 rice samples from various regions of China ranged from 149 to 189 ppb, with cadmium concentrations in 18% of the samples exceeding the normal. Though there has been reports of cadmium contamination in rice from a number of sources, no study has been conducted on the effect of chelating agents in the soaking or/and cooking method on cadmium reduction in rice.

### 3.3 Effect of treatment type on lead removal percentage

As seen in Table 3, there is a statistically significant difference between the various treatments as compared to the control sample, with raw rice reporting the largest level of lead. This value reached 2,877.72 ppb when rice was soaked and cooked without chelating agents, which was still much higher than the standard limit. The use of chelating agents in the elimination or reduction of lead had a significant effect, with the most effect observed in the SC‐Tar and SC‐Cit treatments, but there was no significant difference between the two treatments (*p* > .05). The findings revealed that lead elimination in S‐Tar and C‐Tar treatments differed significantly (*p* < .05) as well as between S‐Cit and C‐Cit. There was a stronger reduction in lead in the cooking process with chelating agents than in the soaking stage with chelating agents (*p* < .05). Since potassium tartrate and citrate salts help metals transfer and migrate from contaminated soils, they may be used as a healthy food additive to help heavy metals migrate from food (Wu et al., 2003). Chelating agents (potassium tartrate and citrate) can effectively remove lead from peanut and canola meal, according to Yang et al. (2016). Furthermore, the removal of heavy metals improved as the concentration of chelating agents was increased, and a linear association was discovered between raising the concentration and increasing the removal efficiency. In general, it can be stated that tartaric acid, citric acid, and their potassium salts can increase the extraction of lead from plant sources due to their carboxyl groups and ability to scavenge metal ions, as shown by previous studies and observations in this study. According to the findings of this study, potassium tartrate outperforms potassium citrate in terms of lead elimination.

**TABLE 3:** Lead and cadmium removal‐percent from dried cooked‐rinsing rice different treatments compared to the blank sample

Treatment	Lead ($\frac{\mu g}{\mathrm{Kg}\;\mathrm{dried}\;\mathrm{rice}}$)	Lead removal (%)	Cadmium ($\frac{\mu g}{\mathrm{Kg}\;\mathrm{dried}\;\mathrm{rice}}$)	Cadmium removal (%)
Blank	3,339.49 ± 851.63^a^	‐	243.33 ± 54.26^a^	‐
SC‐blank	2,877.72 ± 734.14^a^	13.60 ± 1.07^e^	216.81 ± 48.90^a^	12.15 ± 1.23^c^
S‐Tar	242.4 ± 55.34^b^	90.36 ± 0.72^c^	27.78 ± 3.48^b^	81.92 ± 2.01^b^
C‐Tar	141.63 ± 34.45^b^	94.58 ± 0.40^b^	29.99 ± 2.84^b^	79.27 ± 3.09^b^
SC‐Tar	12.79 ± 3.22^b^	99.43 ± 0.15^a^	7.51 ± 1.20^b^	95.13 ± 1.12^a^
S‐Cit	287.17 ± 61.3^b^	87.83 ± 1.02^d^	31.26 ± 3.34^b^	78.43 ± 2.52^b^
C‐Cit	161.05 ± 37.85^b^	92.94 ± 0.69^b^	34.3 ± 3.81^b^	77.61 ± 3.36^b^
SC‐Cit	19.55 ± 4.11^b^	98.96 ± 0.19^a^	10.18 ± 1.43^b^	92.77 ± 1.66^a^

Note

Means ± SE (*n* = 10) with different letters in each column indicate significant difference (*p* < .05). WHO regulations set the lead and cadmium limit for rice at 150 and 60 ppb, respectively.

### 3.4 Effect of treatment type on cadmium removal percentage

Table 3 shows that there is a statistically significant difference between the different treatments and the control sample, with raw rice reporting the highest cadmium level. When rice was soaked and cooked without chelating agents, the content reached 216.81 ppb, which was still much higher than the standard amount. The use of chelating agents in cadmium removal or reduction had a substantial impact, with the greatest effect found in the SC‐Tar and SC‐Cit treatments, but there was no statistical difference between the two treatments (*p* > .05). The results showed that cadmium removal varied not substantially (*p* > .05) between S‐Tar and C‐Tar treatments, as well as between S‐Cit and C‐Cit. Potassium tartrate was used as an efficient removal agent for cadmium extraction from canola meal, resulting in a 75.02 percent cadmium removal efficiency and a reduction in cadmium concentration from 0.95 to 0.24 mg/kg (i.e., 940–240 ppb). They found that increasing the time and temperature of treatment of peanut and canola meal with potassium tartrate and potassium citrate reduced the amount of lead and cadmium more effectively. Huo et al. (2016) used citric acid to remove cadmium from rice. Contaminated rice after treatment with citric acid was used to produce starch and isolate rice protein. Previous research on arsenic reduction found that the soaking and cooking steps had a major impact on reducing the amount of this heavy metal. In contrast to the recorded effects of arsenic reduction in rice cooking, cadmium variations were reported, indicating that the preparation and cooking steps had no impact on reducing cadmium concentration. Also approaches that use a lot of water for rinsing have no impact on cadmium reduction (Chaney et al., 2004; Huo et al., 2016; Khan et al., 2010). After soaking (ratio of distilled water to rice 6:1) and partial cooking of rice (ratio of distilled water to rice 3:1), Mihucz et al. (2007) recorded a cadmium reduction of about 10%–15%. In another study, compared to raw rice, cooked rice (water to rice ratio 1:2) reduced cadmium by 10% (Zhuang et al., 2016). Tartaric acid, citric acid, and their potassium salts, in general, will increase the extraction of cadmium from plant sources due to their carboxyl groups and capacity to scavenge metal ions, as shown by previous studies and findings in this research. In terms of cadmium elimination, potassium tartrate is similar to potassium citrate, according to the results of this study.

### 3.5 Sensory evaluation of pretreated cooked rice

The most significant qualitative parameter is the product's sensory assessment. Table 4 shows the results of sensory assessment of overall acceptance (mean scores of taste, aftertaste, texture, and color) of six varieties of rice cooked under various conditions and in the presence of potassium citrate and potassium tartrate. The sensory evaluation findings of the samples revealed that the treatments had no effect on the overall acceptance of rice, so the panels could not detect variations in different samples treated with chelating salts (*p* > .05). Therefore, the use of relevant salts in addition to reducing heavy metals in rice cannot have an effect on sensory properties to be rejected by the consumer.

**TABLE 4:** Comparison of the treatment type on overall acceptance of cooked‐rinsing rice samples

Treatment	Imported‐rice codes			Exported‐rice codes		
	I	T	U	A	P	S
SC‐blank	4.25 ± 0.69^a^	4.25 ± 0.79^a^	4.20 ± 0.85^a^	4.35 ± 0.69^a^	4.30 ± 0.79^a^	4.25 ± 0.54^a^
S‐Tar	4.10 ± 0.55^a^	4.30 ± 0.69^a^	4.00 ± 0.65^a^	4.20 ± 0.23^a^	4.20 ± 0.33^a^	4.10 ± 0.32^a^
C‐Tar	4.25 ± 0.79^a^	4.35 ± 0.40^a^	4.20 ± 0.56^a^	4.35 ± 0.57^a^	4.25 ± 0.20^a^	4.30 ± 0.36^a^
SC‐Tar	4.00 ± 0.27^a^	4.15 ± 0.93^a^	4.35 ± 0.44^a^	4.01 ± 0.17^a^	4.05 ± 0.73^a^	4.30 ± 0.24^a^
S‐Cit	4.20 ± 0.49^a^	4.45 ± 0.60^a^	4.10 ± 0.72^a^	4.21 ± 0.29^a^	4.35 ± 0.41^a^	4.20 ± 0.42^a^
C‐Cit	4.50 ± 0.61^a^	4.30 ± 0.89^a^	4.20 ± 0.51^a^	4.51 ± 0.41^a^	4.20 ± 0.79^a^	4.30 ± 0.31^a^
SC‐Cit	4.05 ± 0.78^a^	4.10 ± 0.19^a^	4.10 ± 0.39^a^	4.06 ± 0.58^a^	4.00 ± 0.29^a^	4.20 ± 0.19^a^

Note

Means ± SE (*n* = 40) with different letters in each column indicate significant difference (*p* < .05).

## 4 CONCLUSION

Lead and cadmium amounts were higher than the WHO standard in all of the crude rice samples. Cooking than soaking treatments by chelating agents caused more Pb reduction. Cooking than soaking treatments including chelating agents caused to the same Cd reduction. Soaking–cooking treatments including chelating agents significantly reduced Pb, that is, 99%–99.4%. Soaking–cooking treatments including chelating agents significantly reduced Cd, that is, 92.8%–95.1%. Potassium tartrate outperforms potassium citrate in terms of lead reduction. Potassium tartrate is equivalent to potassium citrate in terms of cadmium reduction. Sensory evaluation showed no significant difference between the treatments.

## CONFLICT OF INTEREST

The authors declare that they do not have any conflict of interest, and the study did not involve any human or animal testing.

## AUTHOR CONTRIBUTION

**Zahra Karimi:** Conceptualization (equal); Data curation (equal); Formal analysis (equal); Funding acquisition (equal); Investigation (equal); Methodology (equal); Project administration (equal); Resources (equal); Software (equal); Supervision (equal); Validation (equal); Visualization (equal); Writing‐original draft (equal); Writing‐review & editing (equal). **Mohammad Goli:** Conceptualization (equal); Data curation (equal); Formal analysis (equal); Funding acquisition (equal); Investigation (equal); Methodology (equal); Project administration (equal); Resources (equal); Software (equal); Supervision (equal); Validation (equal); Visualization (equal); Writing‐original draft (equal); Writing‐review & editing (equal).

## Data Availability

The data that support the findings of this study are available from the corresponding author upon reasonable request.
